# Tracing the history of South-American neotropical savannas and seasonally dry forests evidences from comparative phylogeography

**DOI:** 10.1186/1753-6561-5-S7-I3

**Published:** 2011-09-13

**Authors:** Rosane Collevatti, Suelen Rabelo, Joao Nabout, Jose Diniz-Filho

**Affiliations:** 1 Universidade Federal de Goias, Brazil; 2 Universidade Estadual de Goias, Brazil

## Background

The Neotropical Seasonally Dry Forests (SDF) are tree-dominated ecosystems that occur in disjunct areas of fertile soils throughout the Neotropics. The hypothesis that the vicariance of a formerly continuous seasonal woodland formation, which may have reached its maximum extension during a dry-cool period 18,000–12,000 bp (the LGM), the Pleistocenic Arc Hypothesis, has been raised to explain the disjunct distribution [1]. Alternatively, based on the distribution of contemporary SDF species in Amazonia Forest and palynological data, Pennington [2] also proposed that SDF may have expanded into Amazonia Basin during the Pleistoce, with rain forest and montane taxa largely confined to gallery forest. In addition, a number of studies based on the fossil pollen record now available show that during the early Holocene period (until ca. 6000-5000 14C B.P.), the climate was drier in most of the South American savannas and distribution of savanna-like vegetation in Central and Southeast Brazil was more extensive in early compared with the late Holocene [3-5]. In southeastern Brazil, the current vegetation exist in the region only in the latest Holocene period (since 970 or 600 B.P. for some regions) under the current wet climatic conditions, with an annual dry season of about 4 months. Hence, the fossil record shows that savanna expansion in the Quaternary, especially in southeastern Brazil was characterized mainly by herbaceous and grass savanna which were favored by the drier and highly seasonal climate. It is possible that arboreal savanna taxa became restricted to sites with moist climatic conditions, which served as refugias. We are interested in test these hypotheses using the genus *Tabebuia* as model group. We have chosen five species based on the pattern of geographical distribution: *T. aurea* and *T. ochracea*, from savanna vegetation (cerrado sensu stricto), *T. impetiginosa*, T. roseo-alba from SDF, *T. serratifolia*, widely distributed in Mata Atlantica, SDF, riparian forests and Amazonia. Here, we present the results based on the phylogeography of *T. impetiginosa*.

## Methods

We first generated a phylogenetic hypothesis based on sequences from three non-coding regions of cpDNA and ITS from nuclear rDNA. At least 16 individuals from 14 populations were sampled and sequenced for three chloroplast intergenic spacers (1635bp). We also sequenced individuals of *T. ochracea*, *T. chrysanta*, *T. chrysotricha* and *Cybstax antisyphilitica*, as outgroups to estimate coalescence time and better understand the biogeographical history of *T. impetiginosa*. Time to most recent common ancestor was estimated based on coalescent analysis implemented in BEAST 1.4.7 software [6]. For distribution modeling, we used 77 records of *T. impetiginosa* and four climatic variables (mean annual rainfall and variability, average temperature of the warmest and coldest months) derived from four different coupled Atmosperic-Oceanic Global Circulation Models (AOGCM): CCSM3, CSIRO, HADCM3 and ECHAM. Five different niche models were used: the BIOCLIM , Euclidean Distances (EUCL) Mahalanabis Distances (MAHAL), Genetic Algorithm for Rule Set Production (GARP), and Maximum Entropy (MAXENT). For each of the NM, a total of 750 different models were generated, using distinct combinations of dataset partition and variable selection The paleoclimate scenarios were based on the GCM Genesis 2 [7].

## Results and discussion

We have no evidences of a recent connection of the SDF in the LGM. Coalescent analyses showed that *T. impetiginosa* populations probably originated at ~7 Myr BP, but populations started to diverge only ~2 Myr BP, with the divergence of two major clades, one that comprises the populations from the East and West boundaries of Cerrado Biome, and the other that corresponds to the forests from Central, West and Northeast Brazil. Major divergences in this last clade coincide with the Quaternary glaciations. Our results strongly support that the disjunct distribution of *T. impetiginosa* may be derived from vicariance and that long distance gene flow is unlikely, since we found a deep geneanalogy with alopatric lineages and a remarkable differentiation among populations (AMOVA, Arlequin v. 3.11) [8]. Paleodistribution modeling together with coalescent simulation showed no connection among SDFs in the LGM. Thus, glaciations may have most likely caused population divergence and differentiation due to retreat and range shift of an ancient forest widely distributed throughout the Central, Southwest and Northeast Brazil. Hence, we hypothesize that the isolation of an ancient forest due to a global cooling and drying during the glaciations of Pliocene/Pleistocene is responsible for the disjunct distribution of the SDF and *T. impetiginosa*. Our results also indicated that the last glaciation in Pleistocene were most likely responsible for local differentiation because lineage diversification within localities coincides with ~ 100 kya BP but without important demographic effects because populations showed a constant effective population size and no exponential growth or shrink. In conclusion, the range shift of *T. impetiginosa* toward the Northwest and the lack of a connection among SDFs in the LGM support the hypothesis of a kind of SDF in Amazonian Basin during Pleistocene glaciations but did not support the Pleistocenic Arc Hypothesis.
